# Identification of a second encephalitis-associated astrovirus in cattle

**DOI:** 10.1038/emi.2016.5

**Published:** 2016-01-20

**Authors:** Torsten Seuberlich, Daniel Wüthrich, Senija Selimovic-Hamza, Cord Drögemüller, Anna Oevermann, Rémy Bruggmann, Ilias Bouzalas

**Affiliations:** 1 Division of Neurological Sciences, Vetsuisse Faculty, University of Bern, Bern CH-3012, Switzerland; 2 Interfaculty Bioinformatics Unit and Swiss Institute of Bioinformatics, University of Bern, Bern CH-3012, Switzerland; 3 Graduate School for Cellular and Biomedical Sciences, University of Bern, Bern CH-3012, Switzerland; 4 Institute of Genetics, Vetsuisse Faculty, University of Bern, Bern CH-3012, Switzerland

## 

**Dear Editor**,

Human astroviruses are of particular importance as one of the most common pathogens that cause juvenile gastroenteritis. In recent years, several astrovirus isolates were identified as potential causes of encephalitis in immunocompromised human patients.^1,2,3,4,5^ These discoveries were paralleled by the description of phylogenetically closely related bovine astrovirus isolates (BoAstV) from the nervous tissue of cattle with encephalitis of unknown etiology in the USA (BoAstV NeuroS1) and by our laboratory in Switzerland (BoAstV-CH13).^6,7^ BoAstV-CH13 was retrospectively found in approximately one-quarter (5/22) of the cattle with etiologically unresolved non-suppurative encephalitis in Switzerland; however, most of these cases (17/22) were unrelated to BoAstV-CH13. Here, we report the identification of yet another neuroinvasive astrovirus in this set of cattle with non-suppurative encephalitis, which has less than 65% genetic similarity to currently known astrovirus isolates.

In 2006, a 4-year-old Braunvieh cow (case ID 42535) was notified as a clinical bovine spongiform encephalopathy (BSE) suspect to the Swiss authorities. Precise information about the clinical presentation was not available, but the spectrum of signs in BSE-suspect animals usually involves changes in behavior and temperament, hyper-reactivity, and incoordination. Post mortem BSE testing was negative, and histopathological examination led to the diagnosis of severe non-suppurative meningo-encephalomyelitis (Figure 1A). This inflammatory pattern strongly suggested that the animal had a viral infection, but further etiologic investigations were not undertaken. The animal was included in our research on BoAstV infection and encephalitis and was further investigated by unbiased next-generation RNA sequencing (NGS) and a bioinformatics pathogen discovery pipeline.

Illumina sequencing of frozen brain tissue RNA extracts (medulla oblongata) from animal 42 535 resulted in 21 443 420 read pairs. After *in-silico* subtraction of reads that aligned to the bovine reference genome and *de novo* assembly of the remaining reads, we identified four contiguous sequences (contigs) of 792, 1010, 1103, and 3170 nucleotides that matched with the highest amino acid sequence similarity (64%–83%) to different proteins of a sheep astrovirus isolate entry (accession number NC_002469.1) of the National Center for Biotechnology Information database. Gaps between the contigs were bridged by RT-PCR followed by Sanger sequencing. The 5′ and 3′ ends of the RNA molecule were determined by rapid amplification of cDNA ends (Supplementary Methods, Supplementary Table S1). This resulted in a sequence of 6287 nucleotides that revealed features of an astrovirus genome with short 5′ and 3′ untranslated regions, three partially overlapping open reading frames (ORF1a, ORF1b, and ORF2), and a poly-A tail (Figure 1B). RT-PCR targeting a 388-bp fragment in ORF1a confirmed the presence of the viral RNA in frozen tissue samples of the medulla oblongata, cerebellar cortex, midbrain, and cerebral cortex (Supplementary Figure S1). Taken together, these data indicate the presence of a previously unknown astrovirus that we termed BoAstV-CH15 (GenBank accession number KT956903). Other pathogens were not detected using our pipeline.

Full genome phylogenetic comparison placed the BoAstV-CH15 in the same cluster of previously described neurotropic astroviruses (Figure 1C, HMO clade) and distant from bovine and human isolates that were derived from feces specimens (Figure 1C, classical clades). BoAstV-CH15 rooted from the same branch as an ovine astrovirus (OvAstV), which was isolated from the feces of a sheep with diarrhea.^8^ The same topology was obtained in a maximum-likelihood tree that was based on the full-length capsid protein amino acid sequences (Supplementary Figure S2). A sliding-window, pairwise comparison plot of full genome sequences confirmed the relationship between BoAstV-CH15 and the OvAstV and showed less identity of BoAstV-CH15 with BoAstV-CH13 and HuAstV-PS, a human encephalitis isolate, at most positions (Figure 1D).

To assess whether other cases of cattle encephalitis are associated with the presence of the newly identified astrovirus, we tested the entire set of frozen tissue samples (*n* = 22) from the retrospective study mentioned above^7^ using the BoAstV-CH15 RT-PCR protocol. Besides case 42 535, one additional encephalitis case was reactive and showed the specific 388-bp amplicon. This animal was an unrelated neurologically diseased 7-year-old cow that was diagnosed with severe non-suppurative poliomeningoencephalitis and ganglioneuritis in 2007 (ID 42799; Figure 1A). This finding was unexpected in this particular animal because it was previously classified as BoAstV-CH13-positive based on RT-PCR results and ISH experiments.^7^ However, as the BoAstV-CH15 RT-PCR protocol does not detect BoAstV-CH13 (Seuberlich T, 2015, unpubl. data), these conflicting results could be explained by the presence of either a different type of astrovirus or by coinfection with both viruses in the same animal. An RNA extract from the brain tissue of animal 42 799 was therefore similarly subjected to NGS. Mapping the obtained reads to the BoAstV-CH13 and BoAstV-CH15 genomes identified specific reads for 99% of the BoAstV-CH13 genome and 34% of the BoAstV-CH15 genome (Supplementary Figure S3). A BoAstV-CH13-positive control sample (ID 23871)^7^ did not show any reads that mapped to the BoAstV-CH15 genome, which supports that the animal indeed harbored both types of BoAstVs in the brain.

We identified a novel astrovirus (BoAstV-CH15) in the brains of two neurologically affected cows. These findings extend previous reports on neurotropic astrovirus isolates in humans, mink, and cattle.^1,2,3,4,5,6,7,9^ Our results provide evidence that the spectrum of astroviruses associated with non-suppurative encephalitis in cattle is broader than known to this point in time.

In human patients, neurotropic astrovirus isolates revealed ∼95% nucleotide identity with each other, most of them belonged to the HuAstV VA1/HMO-C clade, and they were all identified in immunocompromised patients. It has therefore been postulated that central nervous system invasion occurred via the enteric system as a consequence of immune system impairment.^5^ This notion is supported by the finding that viruses of this clade have also been identified in the feces of a diseased patient^10^ and that there is evidence for a high prevalence of infection in healthy individuals as well.^11^

In bovines, the situation may be different. First, the neurotropic isolates have not yet been identified in feces samples of cattle,^12,13,14^ and second, there is no evidence for an immunocompromised condition in the animals under investigation. The age of the affected cows ranged from 1.5 to seven years, and, thus, the presence of an underlying inherited primary immunodeficiency is unlikely. On the other hand, dairy cows held under intensive production management are often exposed to stressors, such as crowding, pregnancy, lactation, and metabolic imbalances, which may have immunosuppressive effects.^15^ However, information on clinical signs and disease history in these animals is sparse, and any conclusion regarding the disease pathogenesis in cattle would be premature. More comprehensive epidemiological investigations on the clinical presentation, prevalence, and diversity of astrovirus infections in cattle are needed.

The close phylogenetic relationship between animal and human feces and encephalitis isolates within the phylogenetic HMO clade raises an important question concerning the role of cross-species transmission events in astrovirus infection and evolution. Apart from one boy with astrovirus encephalitis living in the vicinity of a mink farm, there have been no clear epidemiological links between astrovirus infections in humans and animals.^1^ The diversity of the HMO clade suggests that transmission between animal species and between animals and humans occurred at different time points in astrovirus evolution. For instance, our data suggest that BoAstV-CH15 originated from a common ancestor with the enteric ovine isolate rather than with bovine enteric isolates. In this sense, future research into astrovirus infections should include activities in both veterinary and human medicine in terms of a ‘one-health' concept.

## Figures and Tables

**Figure 1:**
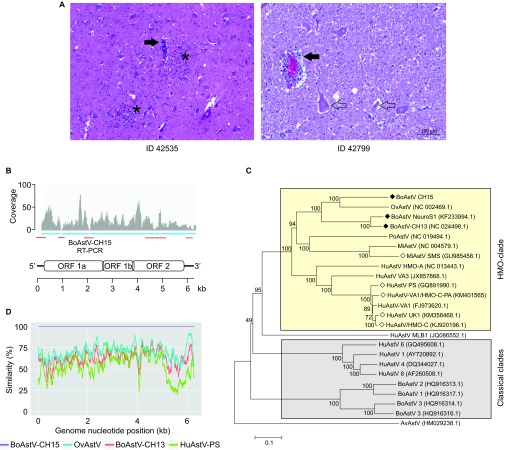
Identification of the novel bovine astrovirus, BoAstV-CH15. (**A**) Representative histopathological lesions in the brains of animal 42 535 (medulla oblongata) and 42 799 (hippocampus). Filled arrows, mononuclear perivascular cuffs; asterisks, glial nodules; open arrows, neuronal necrosis. (**B**) Schematic of the positive strand RNA genome of BoAstV-CH15. The four contigs obtained by NGS and bioinformatics are indicated as blue bars and read coverage is presented in a coverage plot. RT-PCR and Sanger sequencing was performed to bridge the contigs and to determine the 5′ and 3′ ends (red bars). (**C**) Neighbor-joining tree based on full genome nucleotide sequences. BoAstV-CH15 belongs to the human-mink-ovine (HMO) astrovirus clade (yellow box). Filled rhombus, bovine neurotropic strains; open rhombus, neurotropic strains of other species. AvAstV, avian nephritis astrovirus; HuAstV, human astrovirus; MiAstV, mink astrovirus; OvAstV, ovine astrovirus; PoAstV, porcine astrovirus. GenBank accession numbers are noted in brackets. (**D**) Pairwise genome comparison between selected encephalitis-associated astrovirus isolates.

## References

[bib1] 1Quan PL, Wagner TA, Briese T et al. Astrovirus encephalitis in boy with X-linked agammaglobulinemia. Emerg Infect Dis 2010; 16: 918–925. 20507741 10.3201/eid1606.091536 PMC4102142

[bib2] 2Wunderli W, Meerbach A, Güngör T et al. Astrovirus infection in hospitalized infants with severe combined immunodeficiency after allogeneic hematopoietic stem cell transplantation. PLoS One 2011; 6: e27483. 22096580 10.1371/journal.pone.0027483 PMC3214048

[bib3] 3Brown JR, Morfopoulou S, Hubb J et al. Astrovirus VA1/HMO-C: an increasingly recognized neurotropic pathogen in immunocompromised patients. Clin Infect Dis 2015; 60: 881–888. 25572899 10.1093/cid/ciu940 PMC4345817

[bib4] 4Naccache SN, Peggs KS, Mattes FM et al. Diagnosis of neuroinvasive astrovirus infection in an immunocompromised adult with encephalitis by unbiased next-generation sequencing. Clin Infect Dis 2015; 60: 919–923. 25572898 10.1093/cid/ciu912 PMC4345816

[bib5] 5Frémond ML, Pérot P, Muth E et al. Next-generation sequencing for diagnosis and tailored therapy: a case report of astrovirus-associated progressive encephalitis. J Pediatr Infect Dis Soc 2015; 4: e53–e57. 10.1093/jpids/piv040 26407445

[bib6] 6Li L, Diab S, McGraw S et al. Divergent astrovirus associated with neurologic disease in cattle. Emerg Infect Dis 2013; 19: 1385–1392. 23965613 10.3201/eid1909.130682 PMC3810933

[bib7] 7Bouzalas IG, Wüthrich D, Walland J et al. Neurotropic astrovirus in cattle with nonsuppurative encephalitis in Europe. J Clin Microbiol 2014; 52: 3318–3324. 24989603 10.1128/JCM.01195-14 PMC4313157

[bib8] 8Jonassen CM, Jonassen TT, Sveen TM, Grinde B. Complete genomic sequences of astroviruses from sheep and turkey: comparison with related viruses. Virus Res 2003; 91: 195–201. 12573498 10.1016/s0168-1702(02)00269-1

[bib9] 9Blomström AL, Widén F, Hammer AS, Belák S, Berg M. Detection of a novel astrovirus in brain tissue of mink suffering from shaking mink syndrome by use of viral metagenomics. J Clin Microbiol 2010; 48: 4392–4396. 20926705 10.1128/JCM.01040-10 PMC3008476

[bib10] 10Finkbeiner SR, Li Y, Ruone S et al. Identification of a novel astrovirus (astrovirus VA1) associated with an outbreak of acute gastroenteritis. J Virol 2009; 83: 10836–10839. 19706703 10.1128/JVI.00998-09 PMC2753140

[bib11] 11Burbelo PD, Ching KH, Esper F et al. Serological studies confirm the novel astrovirus HMOAstV-C as a highly prevalent human infectious agent. PLoS One 2011; 6: e22576. 21829634 10.1371/journal.pone.0022576 PMC3150362

[bib12] 12Nagai M, Omatsu T, Aoki H et al. Full genome analysis of bovine astrovirus from fecal samples of cattle in Japan: identification of possible interspecies transmission of bovine astrovirus. Arch Virol 2015; 160: 2491–2501. 26212364 10.1007/s00705-015-2543-7

[bib13] 13Sharp CP, Gregory WF, Mason C, Bronsvoort BM, Beard PM. High prevalence and diversity of bovine astroviruses in the faeces of healthy and diarrhoeic calves in South West Scotland. Vet Microbiol 2015; 178: 70–76. 25979841 10.1016/j.vetmic.2015.05.002 PMC4464496

[bib14] 14Tse H, Chan WM, Tsoi HW et al. Rediscovery and genomic characterization of bovine astroviruses. J Gen Virol 2011; 92: 1888–1898. 21508185 10.1099/vir.0.030817-0

[bib15] 15Ingvartsen KL, Moyes KM. Factors contributing to immunosuppression in the dairy cow during the periparturient period. Jpn J Vet Res 2015; 63(S1): S15–S24. 25872323

