# Supplementary material for: Identification of a second encephalitis-associated astrovirus in cattle
Source: Emerg Microbes Infect. 2016 Jan 20;5(1):e5–. doi: 10.1038/emi.2016.5 (PMC4735058; doi:10.1038/emi.2016.5)
Supplement: Supplementary Methods [file emi20165x1.docx]

**Supplementary Methods**

**Nucleic acid extraction.** Approximately 100 μg of frozen brain tissue was collected at the level of the medulla oblongata. For reverse transcription (RT)-PCR analysis, total RNA was extracted with Trizol reagent (Life Technologies) according to the manufacturer’s instructions. For next-generation sequencing, viral RNA was enriched from tissue samples by homogenization, 0.4-µm filtration, and pretreatment with 4 μg RNAse (Promega) and 150 U DNAse (Roche) prior to Trizol extraction. The quality and quantity of purified RNA was assessed by a Bioanalyzer 2100 (Agilent Technologies) and a Qubit® 2.0 (Life Technologies) instrument.

**Next-generation sequencing.** RNA libraries of cases 42535, 42799, and 23871 were prepared with the Illumina’s TruSeq Stranded Total RNA kit (Life Technologies) in parallel with eight other bovine RNA extracts, which were sequenced for different diagnostic purpose. The 10 RNA libraries were then pooled in one lane of an Illumina HiSeq2000, and 2 × 100 bp paired-end reads were collected.

**Bioinformatics pipeline.** Reads of the initial case, 42535, were aligned to the Bos taurus reference genome (version 3.1) using STAR software (version 2.3.0, default options) [1], and unaligned reads were filtered using Trimmomatic (version 0.30, options: SLIDINGWINDOW:4:15 MINLEN:101) [2] . The filtered reads were assembled with SPAdes software (version 3.1.1, options:--sc -k 21, 33, 55, 77, 91, 95, 97, 99) [3]. The resulting assembly was scaffolded using SSPACE software (version 3.0, default options). The scaffolds were aligned to the taxonomic viral uniprot databases, sprot and tremble (18 November 2014), using the NCBI blastx program (version 2.2.29+, default options). Scaffolds that were covered at least 30% with alignments with a similarity of 30% to a viral protein and covered at least 30% of the viral proteins were selected as possible viral sequences. To reduce false positives, the possible viral sequences were aligned to the NCBI non-redundant nucleotide database (5 December 2014) using the blastn program (version 2.2.29+, default options). Scaffolds with an alignment of at least 80 bp to a Bos taurus sequence were excluded.

For the characterizations of case 42799 the reads of this case as well as of cases 42535 and 23871 were mapped to the BoAstV-CH15 and BoAstV-CH13 genomes using Bowtie2 (version 2.2.1) [4]. The read depth was called using GATK (version 3.3.0, option: -T DepthOfCoverage) [5]. The average read depth was calculated in a sliding window with the length of 1 % of the genomes.

**Sanger sequencing and RACE.** Total RNA was reverse transcribed to cDNA with the ThermoScript® reverse transcriptase (Life Technologies) using oligo dT and gene-specific primers. To bridge the gaps between contigs, the cDNA was amplified with GoTaq® G2 Green Master Mix (Promega) and combinations of primers that mapped to at least 60 nucleotides up- and downstream of the gaps (Supplementary Table 2). The 5’ RACE and Gene Racer kits (Life Technologies) were used to determine the 5’ and 3’ ends of the viral genome. Reactions were carried out in a GenAmp PCR System 9700 (Applied Biosystems) in a volume of 50 μl with the amplification conditions: 94°C for 5 min; 40 cycles of 95°C for 30 sec, 55°C for 30 sec, and 72°C for 30 sec; and a final step at 72°C for 5 min. Amplicons were purified with QIAquick PCR Purification kits (QIAGEN) and directly sequenced in both directions.

**BoAstV-CH15 RT-PCR.** RNA extracts of frozen brain tissues from 22 cases of non-suppurative encephalitis as well as extracts from different brain regions of case 42535 were analyzed with the primers, BoAstV-CH15 3 do and BoAstV-CH15 3 fo, in a one-step protocol (OneTaq® One-Step, New England Biolabs) using the following cycle parameters: 48°C for 15 min; 94°C for 1 min; 40 cycles of 94°C for 15 sec, 52°C for 30sec, and 68°C for 45 sec; and 68°C for 5 min. PCR products were analyzed by electrophoresis in 1% (w/v) agarose gels.

**Phylogenetic analysis.** A neighbor-joining tree based on the full genome nucleotide sequences of representative *Astroviridae* members was constructed using MEGA5 software (http://www.megasoftware.net/) and the Jukes-Cantor model with 1,000 bootstrap replicates. Based on the full-length capsid protein amino acid sequences, a maximum-likelihood tree using a Jones-Taylor-Thornton model and 1,000 bootstrap replicates was generated to assess whether the topology was associated to specific open reading frames of the virus. Full genomes of three representative *Astroviridae* members along with the new BoAstV-CH15 genome were aligned using MEGA5. The similarity was calculated for every position of the BoAstV-CH15 genome using a sliding window of 100-bp length. The results were plotted using the R package, ggplot2 [6].

**References**

[1] A. Dobin, C. a. Davis, F. Schlesinger, J. Drenkow, C. Zaleski, S. Jha, P. Batut, M. Chaisson, and T. R. Gingeras, “STAR: Ultrafast universal RNA-seq aligner,” Bioinformatics, vol. 29, no. 1, pp. 15–21, 2013. [https://github.com/alexdobin/ STAR/releases]

[2] A. M. Bolger, M. Lohse, and B. Usadel, “Trimmomatic: a flexible trimmer for Illumina sequence data.,” Bioinformatics, vol. 30, no. 15, pp. 2114–2120, Apr. 2014. [http://www.usedellab.org/cms/?Page=trimmomatic]

[3] A. Bankevich, S. Nurk, D. Antipov, A. a Gurevich, M. Dvorkin, A. S. Kulikov, V. M. Lesin, S. I. Nikolenko, S. Pham, A. D. Prjibelski, A. V Pyshkin, A. V Sirotkin, N. Vyahhi, G. Tesler, M. a Alekseyev, and P. a Pevzner, “SPAdes: a new genome assembly algorithm and its applications to single-cell sequencing.,” J. Comput. Biol., vol. 19, no. 5, pp. 455–77, May 2012. [http://spades.bioinf.spbau.ru/release3.1.1]

[4] B. Langmead and S. L. Salzberg, “Fast gapped-read alignment with Bowtie 2.,” Nat. Methods, vol. 9, no. 4, pp. 357–9, Apr. 2012.

[5] A. McKenna, M. Hanna, E. Banks, A. Sivachenko, K. Cibulskis, A. Kernytsky, K. Garimella, D. Altshuler, S. Gabriel, M. Daly, and M. a DePristo, “The Genome Analysis Toolkit: a MapReduce framework for analyzing next-generation DNA sequencing data.,” Genome Res., vol. 20, no. 9, pp. 1297–303, Sep. 2010.

[6] H. Wickham, “ggplot2: Elegant Graphics for Data Analysis,” J. Stat. Softw., vol. 35, no. July, pp. 1–3, 2010.
