# Supplementary material for: Identification of a second encephalitis-associated astrovirus in cattle
Source: Emerg Microbes Infect. 2016 Jan 20;5(1):e5–. doi: 10.1038/emi.2016.5 (PMC4735058; doi:10.1038/emi.2016.5)
Supplement: Supplementary Figures and Table [file emi20165x2.pdf]

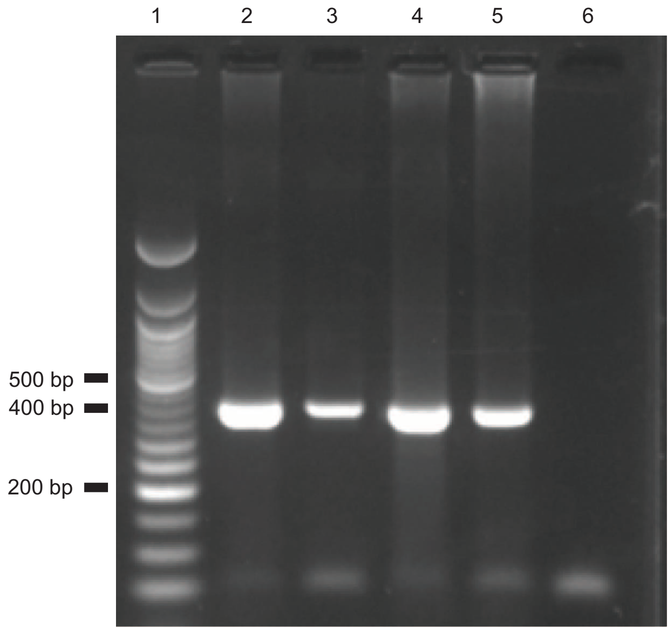

Lane 1: Molecular marker 50 bp (New England Biolabs)

Lane 2: Case 42535, medulla oblongata

Lane 3: Case 42535, cerebellum

Lane 4: Case 42535, midbrain

Lane 5: Case 42535, cerebral cortex

Lane 6: No template control

Supplementary Figure S1 Detection of BoAstV-CH15 RNA in different brain regions of case 42535 by BoAstV-CH15 RT-PCR. The virus was detected in all of the examined brain regions (medulla oblongata, cerebellum, midbrain, and cerebral cortex).

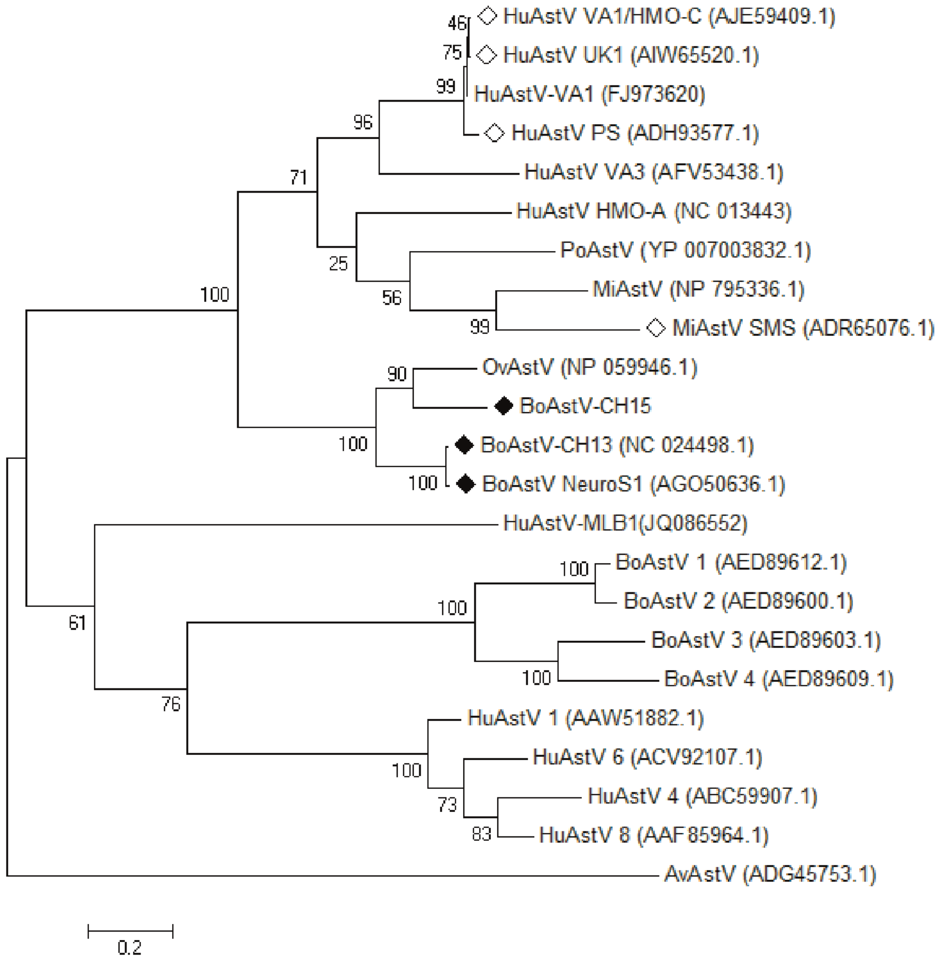

Supplementary Figure S2 Maximum-likelihood tree constructed from amino acid sequences of the full-length capsid protein. GenBank accession numbers are given in the brackets. AvAstV, avian nephritis virus; BoAstV, bovine astrovirus; HuAstV, human astrovirus; MiAstV, mink astrovirus; OvAstV, ovine astrovirus; PoAstV, porcine astrovirus. Filled rhombus, bovine neurotropic strains; open rhombus, neurotropic strains from species other than bovine.

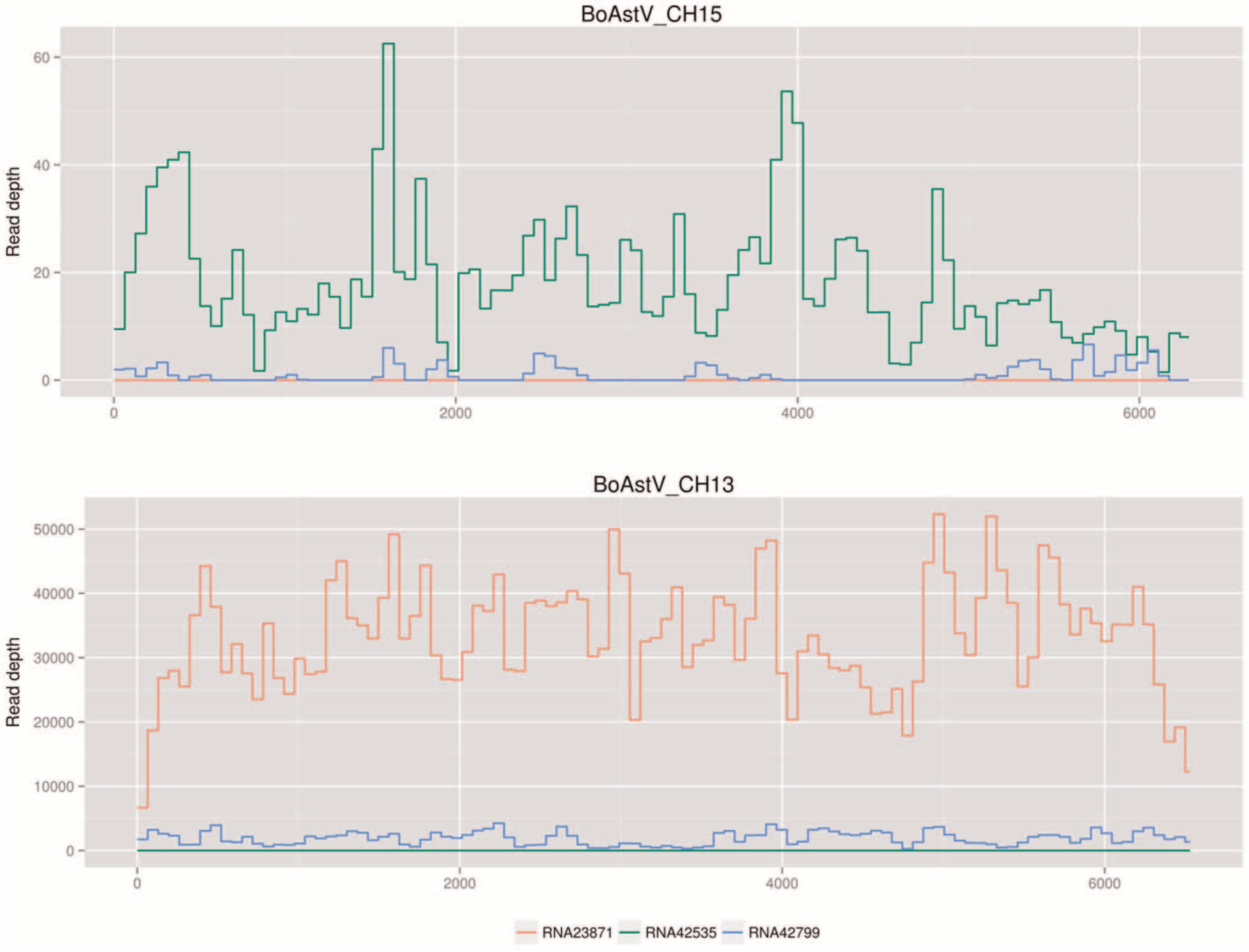

Supplementary Figure S3 Mapping of next-generation sequencing reads from cases 42 535, 42 799, and 23 871. Cases 42 535 (BoAstV-CH15 positive), 42 799 (presumptive BoAstV-CH15/BoAstV-CH13 coinfection), and the BoAstV-CH13-positive control sample, 23 871, were compared to BoAstV-CH15 (upper panel) and BoAstV-CH13 (lower panel) genomes. The *x*-axis indicates nucleotide positions in the reference genomes.

Supplementary Table 1. Primers used for completing the gaps between contigs derived from next-generation sequencing and for BoAstV-CH15 RT-PCR.

Primer Code	Sequence (5'-3')	Orientation	Binding site	Application
BoAstv 15 1do	TGG GAG AAC TCC AGC AAC	−	100–83	5' RACE nested
BoAstv 15 2fo	CAG CAA CGG TTG GTC TTT G	+	738–756	bridge 1st and 2nd contigs
BoAstv 15 2do	GTC CCT TGA CCA TTG TTG C	−	884–866	bridge 1st and 2nd contigs
BoAstV 15 3fo	GTC TTG CGC GCT GAG C	+	1757–1772	bridge 2nd and 3rdcontigs
BoAstV 15 3do	TGG GTA ATT CTC TAA GCT GTA CTT	−	2145–2123	bridge 2nd and 3rd contigs
BoAstV 15 4fo	ATG GCC CGC CAT ATG G	+	4805–4820	bridge 3rd and 4th contigs
BoAstV 15 4do	CAA GGT ATC TAA CGT GTA CCA C	−	5440–5419	bridge 3rd and 4th contigs
BoAstV 15 5fo	GAA TGC CTG CGT AGC ACC	+	6101–6118	3' RACE
